# Prenatal Exposure to Emerging Pesticides and Childhood Allergy Risk: A Mixture Assessment in an Urban Birth Cohort

**DOI:** 10.1021/acs.estlett.5c00836

**Published:** 2025-11-17

**Authors:** Sergio Gómez-Olarte, Stefan Röder, Michael Borte, Martin Krauss, Werner Brack, Ana C. Zenclussen, Gunda Herberth, Carolin Huber

**Affiliations:** † Department of Environmental Immunology, 28342 Helmholtz Centre for Environmental Research−UFZ, Leipzig 04318, Germany; ‡ St. Georg Hospital Leipzig, Department of Pediatrics, Leipzig 04129, Germany; § Department of Exposure Science, Helmholtz Centre for Environmental Research−UFZ, Leipzig 04318, Germany; ∥ Department of Evolutionary Ecology & Environmental Toxicology, Faculty of Biological Sciences−Goethe University Frankfurt, Frankfurt am Main, 60438, Germany; ⊥ German Center for Child and Adolescent Health (DZKJ), Partner Site Leipzig/Dresden 04103, Germany; # Perinatal Immunology, Saxonian Incubator for Clinical Translation (SIKT), Medical Faculty, Leipzig University, Leipzig 04103, Germany

**Keywords:** asthma, eczema, wheezing, pesticide metabolites, weighted quantile sum (WQS) regression

## Abstract

Pesticide gestational exposure may contribute to the development of allergies in childhood, yet evidence on its health impact on urban populations remains limited. This study investigates the association between prenatal exposure to individual and mixed pesticides and allergic outcomes, including asthma, wheezing, and eczema, at age 6 in 387 mother-child pairs from the German prospective cohort LiNA. Forty pesticides and metabolites were detected in urine during pregnancy through nontargeted screening, and 11 were selected (detection rate ≥ 17%) for further analysis. Multivariable logistic regression models adjusted for covariates revealed statistically significant associations between dihydroxy-pyrimethanil and asthma (aOR = 1.35, 95% CI: 1.02–1.79), and fluazifop-desbuthyl and wheezing (aOR = 1.14, 95% CI: 1.01–1.30). No significant associations were observed for eczema. The weighted quantile sum (WQS) regression showed that higher pesticide coexposures significantly increased wheezing odds (aOR = 2.08, 95% CI: 1.21–3.56). The main components of the WQS index were fluazifop-desbuthyl, flonicamid, hydroxy-metazachlor, and terbuthylazine, accounting for 67% of the overall mixture effect. These findings suggest that prenatal exposure to pesticides, likely from dietary sources, may increase the risk of childhood asthma and wheezing. Replication studies in populations with comparable pesticide exposures, along with experimental mechanistic validation, will strengthen the understanding of the observed associations.

## Graphical abstract

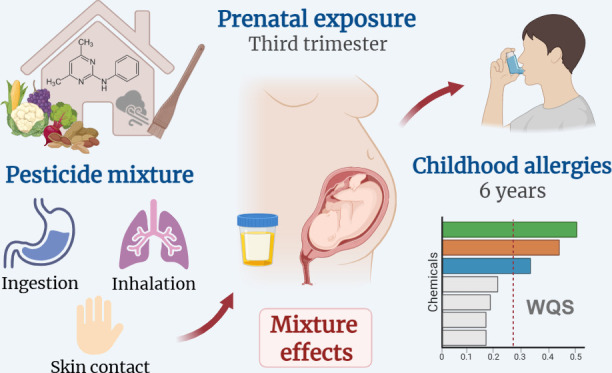

## 1 Introduction

Some of the pesticides introduced during the Green Revolution were listed as persistent organic pollutants, including organochlorine pesticides,^1^ and phased out by the 2000s due to growing health concerns for pregnant women and children.^2^ For instance, a meta-analysis of seven European cohorts showed that prenatal exposure to the main dichlorodiphenyltrichloroethane (DDT) metabolite and polychlorinated biphenyl (PCB)-153 was associated with bronchitis and wheezing in children aged 4 years old.^3^ This suggests that gestation is a key window of susceptibility to pesticide exposure, as the fetal immune system is still in the developing phase.^4^

Gradually, persistent pesticides have been replaced with less persistent target-specific compounds, such as carbamates and neonicotinoids.^5^ Despite modern pesticides displaying an abridged human toxicity, scarce evidence indicates they could be associated with the development of allergic disorders early in life.^6^ In particular, the Costa Rican ISA cohort and a French pilot study reported an association of current exposure to pyrethroid and mancozeb (a carbamate pesticide) metabolites with asthma at ages 3–10 years,^7–9^ respectively.

Most epidemiological evidence about pesticide effects on allergic outcomes derives from populations living in rural farming areas, where maternal exposure is at least five times higher than in the general public.^10^ Therefore, it does not reflect the health risks in urban environments, in which pesticide exposure primarily occurs through diet at lower doses.^11^ In this regard, a New York cohort found that prenatal exposure to the insecticide permethrin increased the odds of cough, but not asthma or wheezing, in children 5–6 years old.^12^ Recently, the ELFE cohort explored how urban pesticide coexposures during pregnancy might contribute to childhood allergies. Although this longitudinal study linked intake of foods containing pesticide mixtures to wheezing risk at age 5.5,^13^ it overlooked potential mixture effects that could enhance the overall toxicity.

The present study aims to investigate whether prenatal exposure to pesticides and their metabolites is associated with the onset of asthma, wheezing, and eczema in preschool children from the LiNA cohort. Pesticides can cross the placental barrier and may disrupt fetal immune development, prompting the development of atopic disorders later in life.^4,14^ Thus, we addressed the health effects of 11 pesticides with a focus on the potential mixture effects of coexposures.

## 2 Materials and Methods

### 2.1 Study Design and Recruitment

The Lifestyle and environmental factors and their influence on the Newborn Allergy risk (LiNA) prospective birth cohort recruited 622 pregnant women between 2006 and 2008 in Leipzig, Germany, under the approval of the Review Board of Leipzig University (No. 046-2006, Supporting text 1-2). All participants signed the informed consent voluntarily. This study comprises 581 mothers who provided urine samples and questionnaire information at gestational week (WG) 34–36, including occupation, and their paired children aged 6 years. Statistical analyses were performed in 387 mother-child pairs after excluding those lost to follow-up, twins, and participants without exposure assessment (Figure S1).

### 2.2 Chemical Exposure Assessment

Pesticides and their metabolites were detected in first morning void urine samples (third trimester of pregnancy), which were collected and stored in polypropylene tubes at −80 °C until analysis. After thawing, 500 μL aliquots were used for sample preparation and measurement using liquid chromatography-high-resolution mass spectrometry (LC-HRMS), as previously described.^15^ The following pesticides or corresponding metabolites detected in at least n = 100/581 mothers (detection rate: DR ≥ 17%) were selected for the health assessment: metalaxyl, carbetamide, terbuthylazine, imidacloprid, flonicamid, hydroxy-isoproturon, dihydroxy-pyrimethanil, hydroxy-simazine, hydroxy-propamocarb, fluazifop-desbuthyl, and hydroxy-metazachlor. The nontargeted measurements provided uncorrected peak intensities, referred to as levels onward, but not precise chemical concentrations. When more than one marker of a pesticide was available, the parent compound or metabolite with the highest DR measurement was implemented in the regression models.

### 2.3 Allergic Outcomes and Covariates

Information on childhood allergies and related symptoms (asthma, wheezing, and eczema) was collected annually via standardized self-reported questionnaires, asking the parents for a doctor’s diagnosis in the previous 12 months. Parental reports up to age 6 were used to define the lifetime prevalence of the outcomes. General sociodemographic and lifestyle factors were retrieved from questionnaires completed during the third trimester of pregnancy and annually after the child’s birth. Among these variables, smoking/environmental tobacco smoke (ETS) exposure during pregnancy, breastfeeding throughout the first 6 months, family history of atopy, parental education level, and child sex were identified as potential covariates based on previous literature^16^ (see directed acyclic graph, Figure S2).

### 2.4 Statistical Analysis

Given right-skewed distributions, chemical peak intensities were log_2_-transformed for regression analysis and presented using the geometric mean (GM) and percentiles. Mother-child pairs’ characteristics and covariates were described with numbers (n) and percentages (%) and compared among pregnancy and 6-year follow-up observations using a Chi-square or Fisher’s exact test (*n* < 5). Statistical significance was defined with a *p*-value threshold of 0.05.

Individual associations between 11 pesticides detected in the mothers (DR ≥ 17%) and the children’s outcomes were investigated using multivariable logistic regression models, crude and adjusted for covariates. Missing data for smoking (n = 2) and breastfeeding (n = 11) were completed using multiple imputation (MI, n = 20) by chained equations with logistic regression models. Exposure values below the detection limit (BDL) were imputed (n = 20) via a truncated log-normal function as recommended before,^17^ setting the lowest measured peak intensity value (limit of detection: LOD) divided by √2 as the compound-specific detection threshold. The regression models were fitted with each of the 20 imputed data sets, and the resulting estimates were pooled using Rubin’s rules to account for missing data uncertainty.^18^ Another exposure data set was created by imputing BDL values with LOD/√2 and used for sensitivity analysis. Human pesticide coexposure is linked to common sources like food, often producing exposure matrices with correlated components.^19^ Hence, we explored the collinearity between the pesticides and metabolites through Spearman’s rank correlation matrices of the original, MI-imputed, and LOD/√2-imputed data sets.

Furthermore, multipollutant analyses accounting for potential mixture effects among the pesticides were conducted using weighted quantile sum (WQS) regression.^20^ Pesticide measurements were categorized into three quantiles (Qs); values BDL were assigned to Q1 (nonexposed/low exposure), while those detected were scored to Q2 and Q3 (medium and high exposures). This strategy offers the best model fit, accuracy, and statistical power to identify chemical mixture effects when exposure values BDL are 50–80%,^21^ as in our pesticide exposure data. When fitting the crude and adjusted WQS with each allergy outcome, the input data were randomly split into training and validation sets (0.40 and 0.60) to estimate single chemical weights by the bootstrap method (n = 200) and the repeated holdout validation procedure (n = 20).^22^ Pesticides with weights >9% were classified as the main drivers of mixture effects (cutoff τ value = 1/n, n = 11).^20^

To assess exposure risk, nonadjusted and adjusted odds ratios (OR/aOR) and 95% confidence intervals (CIs) were estimated from logistic regression coefficients, with values >1.0 indicating increased risk. Statistical analyses and plots, including MI, correlation matrices, and WQS regression models, were computed with the R software (v4.4.1) using the packages “mice” (v3.18),^23^ “corrplot” (v0.95),^24^ and “gWQS” (v3.0.5).^25^

## 3 Results and Discussion

### 3.1 Sociodemographic Characteristics, Allergic Outcomes, and Pesticide Exposure Levels

The present study included a subgroup of 387 mother-child pairs, whose sociodemographic and lifestyle features were similar (*p* > 0.05) to the entire LiNA cohort with pesticide exposure assessment during pregnancy (n = 581) (Table S1). The lifetime prevalence of asthma, wheezing, and eczema in the 6-year-old children was 4.9% (n = 19), 43% (n = 167), and 22% (n = 87), respectively. Other cohorts assessing similar pesticide exposures have reported a higher prevalence of asthma (12% and 7.7%) and a comparable or lower prevalence of wheezing (40% and 32%) in 5-year-old children.^7,13^ The prevalence of eczema in children from these studies was markedly lower (7%) or about double (48%) that in the LiNA cohort (22%).^7,13^ These differences might stem from regional, methodological, or diagnostic variations among the studies.

Nontargeted analyses revealed that children were prenatally exposed to mixtures of pesticides and their derived metabolites, which are commonly used as fungicides, herbicides, and insecticides (Figure 1A). The mothers did not report occupational pesticide exposure; thus, dietary intake was the most likely chemical source, as demonstrated by our analysis of polyphenol food markers.^15^ The distribution levels of pesticides and metabolites are shown in Table S2. Findings were mostly consistent across the three exposure matrices (Figure 1B/Figure S3), with positive (*r* ≥ 0.64; *p* < 0.05) correlations between metalaxyl and carbetamide, carbetamide and hydroxy-isoproturon, and metalaxyl and hydroxy-isoproturon. Since it has been shown that assigning truncated random MI values to measures BDL generates unbiased regression parameter estimates,^17^ we employed the MI-imputed data set (log_2_-transformed) to fit the multivariable regression models.

**Figure 1:**
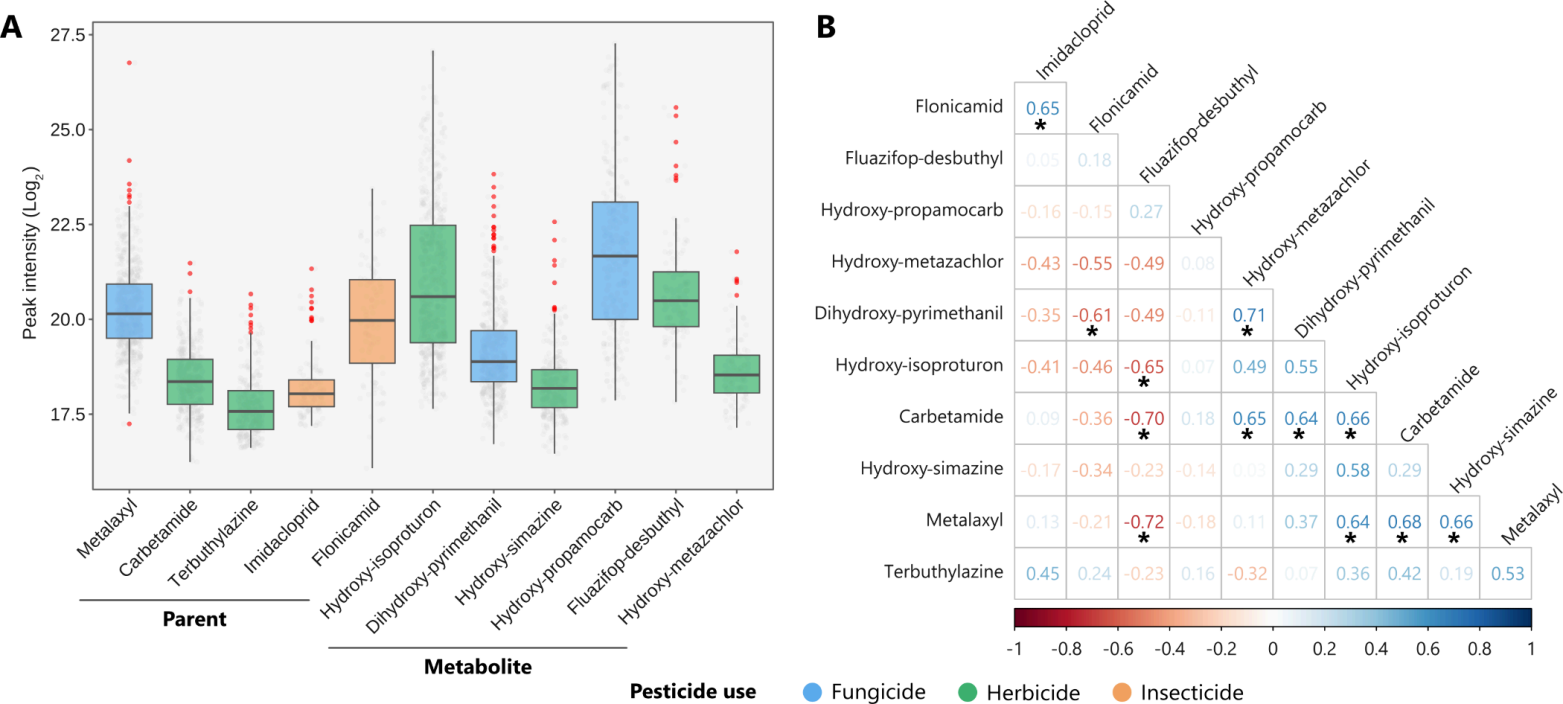
(A) Box plot that displays the distribution of pesticides and metabolites measured (log_2_-transformed) in urine during pregnancy (WG 34–36); red dots denote extreme values. Chemicals are shown from left to right according to their type (parent or metabolite) and DR (highest to lowest). Note that peak intensities do not allow comparing concentrations among the analyzed pesticides; they simply reflect the LC-HRMS instrument response. (B) Pairwise Spearman’s rank correlation matrix of chemical exposure levels, where values BDL were imputed with MI (*n* = 20). In the color spectrum, blue and red shades show positive and negative correlations between the chemicals. The asterisk (*) denotes statistically significant correlations (*p* < 0.05).

### 3.2 Association of Pesticides and Metabolites with Allergic Outcomes

We investigated the association between the individual pesticides (parent, n = 5; metabolites, n = 6) and allergic outcomes, including asthma, wheezing, and eczema, in 6-year-old children by multivariable logistic models after controlling for covariates (Figure S2). The precise ORs, 95% CI, and *p*-values for all estimates are depicted in Table S3. Both the crude and adjusted models showed a statistically significant positive association of dihydroxy-pyrimethanil with asthma (aOR = 1.35, 95% CI: 1.02–1.79) and fluazifop-desbuthyl with wheezing (aOR = 1.14, 95% CI: 1.01–1.30); whereas, there was no relationship between the chemical exposures and eczema (Figure 2). Thus, prenatal exposure to doubling levels of pyrimethanil and fluazifop metabolites increased the odds of asthma and wheezing by 35% and 14% in children 6 years old, respectively. Sensitivity analysis with the LOD/√2-imputed exposure data set consistently showed positive associations (*p* < 0.05) and ORs of similar magnitude between dihydroxy-pyrimethanil and asthma, and fluazifop-desbuthyl and wheezing (Table S4).

**Figure 2:**
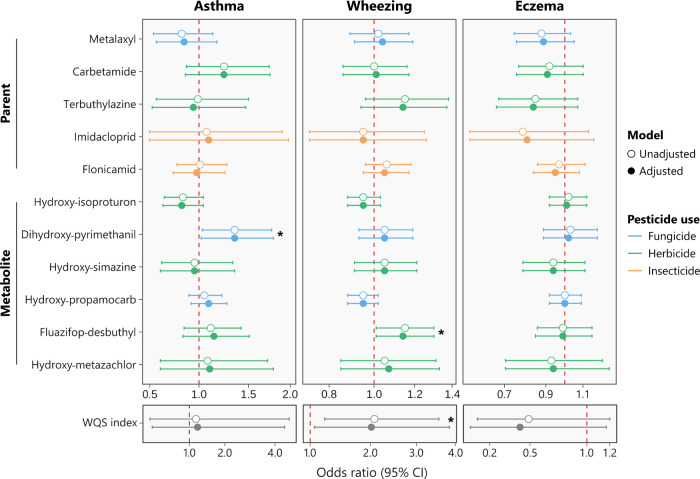
Forest plot of the association between prenatal exposure to 11 pesticides (single compounds and WQS index) and the allergic outcomes asthma, wheezing, and eczema in 6-year-old children (*n* = 387). The logistic regression models were adjusted for smoking/ETS exposure during pregnancy, breastfeeding up to 6 months, parental atopy history, parental education level, and child sex. Chemicals are shown top-down according to their type (parent or metabolite) and DR (highest to lowest). The precision of the estimates is indicated using 95% CIs. The asterisk (*) denotes statistically significant associations (*p* < 0.05).

Several epidemiological studies have reported positive associations of prenatal pesticide exposure, mostly to DDT and organophosphates, with respiratory^26–31^ and allergic^16,32^ outcomes. However, only a few of them assessed^7,8,13,33^ the exposure to the pesticides detected in the LiNA cohort. Comparisons across these findings are challenging due to differences in population exposure levels, detected chemical forms, and the timing of outcome assessment. For instance, the Costa Rican ISA cohort did not find a relationship between prenatal levels of hydroxy-pyrimethanil and wheezing during the first year of life,^8^ which aligns with our observation of no association between dihydroxy-pyrimethanil and wheezing at age 6. This showcase concordant results for an outcome using two different metabolites of pyrimethanil at infancy and preschool age; yet, we found that dihydroxy-pyrimethanil was linked to asthma. Interestingly, LiNA urine samples that contained polyphenol markers for citrus fruit consumption featured higher DRs of pyrimethanil metabolites.^15^

Next, we assessed whether prenatal exposure to the pesticide mixture was associated with allergic outcomes in the 6-year-old children using WQS regression. The WQS models of asthma, wheezing, and eczema were adjusted for covariates and restricted to the positive index to examine potential mixture effects among the exposures. The pesticide WQS index exhibited a statistically significant positive association (β=0.70, 95% CI: 0.06–1.34) with wheezing. This indicates that a one-quantile increase (from low to medium and medium to high exposure levels) in the WQS index doubled the odds of wheezing in children aged 6 years (aOR = 2.08, 95% CI: 1.21–3.56). On the contrary, the relationships between the pesticide WQS index and asthma (dihydroxy-pyrimethanil weight = 15%) or eczema were not statistically significant (Figure 2). The main contributors to the overall mixture effect on wheezing were fluazifop-desbuthyl (26%), flonicamid (19%), hydroxy-metazachlor (11%), and terbuthylazine (11%), which had a combined weight of 67% (Figure 3). The WQS index could capture the modest contribution of the latter three chemicals, which may be missed when each pesticide’s effect is only estimated with single regression models.^34^ As of now, scarce toxicological studies have investigated the potential mechanisms linking prenatal exposure to these pesticides with the development of asthma and wheezing. For instance, fluazifop-butyl has been shown to induce oxidative stress and apoptosis in mouse testis cells *in vitro*,^35^ whereas terbuthylazine exposure triggered myocardial damage in broiler chickens via activation of the cGAS-STING/NF-κB proinflammatory pathway.^36^ This might be indirect evidence for a putative role of the identified pesticides of concern in driving inflammation and atopic disorders through immune-related mechanisms.^37,38^

**Figure 3:**
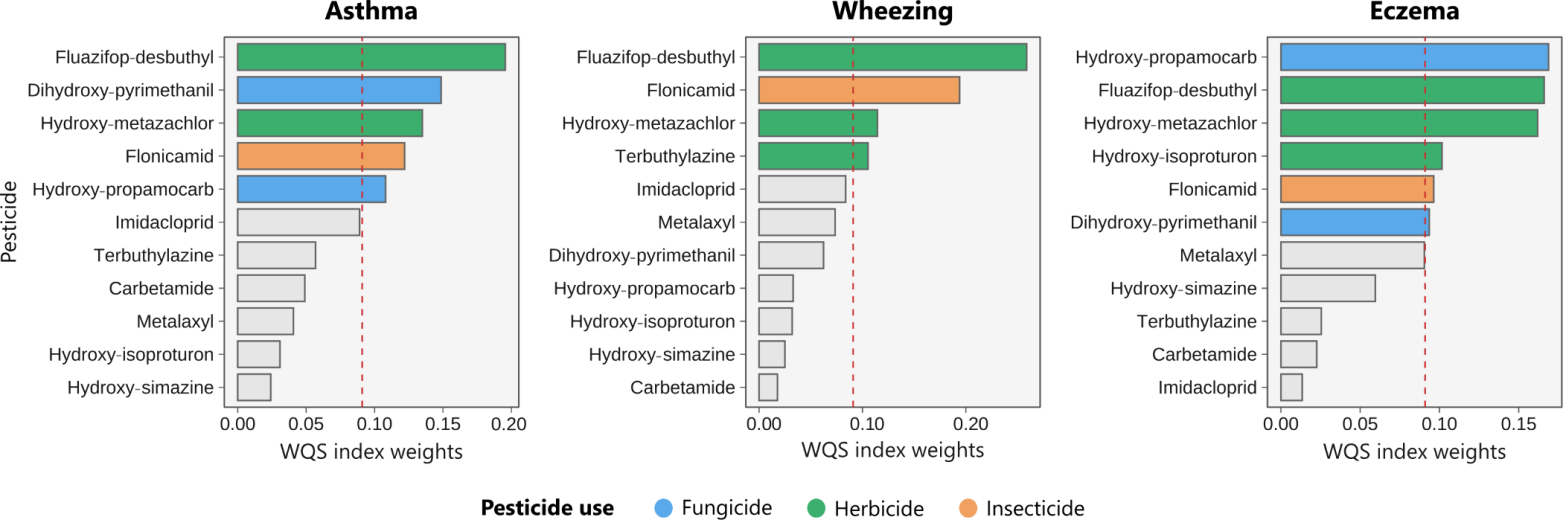
Weight magnitudes from the WQS index for the association of pesticides and metabolites with asthma, wheezing, and eczema prevalence in 6-year-old children. Estimates of the WQS regression were adjusted for smoking/ETS exposure during pregnancy, breastfeeding up to 6 months, parental atopy history, parental education level, and child sex. The dashed red lines represent the cutoff τ (inverse of exposure variables = 1/*n*, *n* = 11) that defines the exposures driving the effect within the chemical mixture.

To our knowledge, only recent studies from the ELFE and EDEN cohorts have investigated pesticide mixture effects during early life.^13,33^ The ELFE study reported an increased risk of wheezing in 5.5-year-old children who were prenatally exposed to high levels of mixed trace elements and pesticides (pyrimethanil, metalaxyl, and imidacloprid) detected in the mother’s diet.^13^ Although these studies featured a robust sample size, the mixture analysis relied on exposure scores derived from linking nutritional questionnaires to pesticide measurements in foods, which restricted the accuracy of the exposure assessment.^13,33^ Notably, the Green Housing multicity cohort examined the association between current exposure to a pesticide metabolite mixture and asthma outcomes in a small sample (n = 162) of children ages 7–12 years and found no relationship.^39^ The current work provides novel evidence on how mixture effects of prenatal pesticide coexposures, at nonoccupational levels, may contribute to the development of childhood wheezing. Moreover, our findings and other studies suggest that routine chemical monitoring should consider pesticide metabolites as markers of internal exposure, especially for the risk assessment of chemical mixtures.^40^

### 3.3 Strengths and Limitations

This study has several strengths. LiNA cohort’s longitudinal and prospective design could support a potential causal relationship between prenatal exposure to a pesticide mixture and wheezing risk. By implementing appropriate WQS mixture modeling, we could identify the major chemicals, three herbicides and one insecticide, driving this mixture effect. The robustness of the regression estimates was tested by fitting crude and adjusted models with exposure data sets completed with MI and fixed (LOD/√2) values. Thus, in contrast to most studies, we considered exposures with about 20% of detected values in the analyses and found they may contribute to chemical cocktail effects. In particular, it shows that metabolites can have higher DRs than parent compounds, making them more reliable for exposure assessment.^41,34^ Nonetheless, the present study also has limitations related to the chemical assessment and children’s outcome prevalence. As pesticides were measured in a unique random urine sample during pregnancy, we may have overlooked pesticides or other chemicals with short half-lives, such as carbamates, or residual confounding due to postnatal pesticide exposures. The nontargeted screening only provides signal intensity values that correlate with the concentration of the pesticides and metabolites. This limits our capability to compare exposure levels with those of other populations. Besides, the collection of allergic outcome information by self-reports, although with standardized questionnaires, has the potential for recall and misclassification biases. The low prevalence of asthma (<5%) in the LiNA cohort at age 6 may have restricted the statistical power of the regression models to detect modest or weak associations with low-DR pesticides. Therefore, we recommend conducting replication studies in larger populations with comparable exposure patterns and experimental bioassays to explore the underlying mechanisms.

## Supplementary Material



## Notes

General descriptions of the LiNA study and the City of Leipzig, summary of sociodemographic and lifestyle characteristics of LiNA cohort mother-child pairs, distribution of pesticide and metabolite peak intensities in urine samples of LiNA mother-child pairs at age 6, output from logistic regression models for prenatal exposure to pesticides and allergies in 6-year-old children (*n* = 387), using the exposure data sets imputed with MI and LOD/√2, flowchart of recruitment and inclusion criteria of LiNA mother-child pairs at age 6, directed acyclic graph that displays the causal pathway linking pesticide prenatal exposure to allergic outcomes, and Spearman’s rank correlation matrices of chemical exposure levels for the original and LOD/√2-imputed data sets (PDF)
