# Supplementary material for: Prenatal Exposure to Emerging Pesticides and Childhood Allergy Risk: A Mixture Assessment in an Urban Birth Cohort
Source: Environ Sci Technol Lett. 2025 Nov 17;12(12):1611–7. doi: 10.1021/acs.estlett.5c00836 (PMC12879297; doi:10.1021/acs.estlett.5c00836)
Supplement: Supplementary file 1 [file ez5c00836_si_001.pdf]

Supporting Information

Prenatal Exposure to Emerging Pesticides and Childhood Allergy Risk: A Mixture Assessment in an Urban Birth Cohort

Sergio Gómez-Olarte [a, *], Stefan Röder [a], Michael Borte [b], Martin Krauss [c], Werner Brack [c, d], Ana C. Zenclussen [a, e, f], Gunda Herberth [a, e, *, #], Carolin Huber [c, #]

Affiliations

[a] Department of Environmental Immunology, Helmholtz Centre for Environmental Research – UFZ, Leipzig, 04318, Germany
[b] St. Georg Hospital Leipzig, Department of Pediatrics, Leipzig, 04129, Germany
[c] Department of Exposure Science, Helmholtz Centre for Environmental Research – UFZ, Leipzig, 04318, Germany
[d] Department Evolutionary Ecology & Environmental Toxicology, Faculty of Biological Sciences – Goethe University Frankfurt, Frankfurt am Main, 60438, Germany
[e] German Center for Child and Adolescent Health (DZKJ), Partner Site Leipzig/Dresden, 04103, Germany
[f] Perinatal Immunology, Saxonian Incubator for Clinical Translation (SIKT), Medical Faculty, Leipzig University, Leipzig, 04103, Germany
[#] *GH and CH contributed equally as last authors*

*** Corresponding Authors**

Gunda Herberth, PhD. Department of Environmental Immunology, Helmholtz Centre for Environmental Research – UFZ, Permoserstraße 15, 04318, Leipzig, Germany. E-mail: gunda.herberth@ufz.de
Sergio Gómez-Olarte, PhD. Department of Environmental Immunology, Helmholtz Centre for Environmental Research – UFZ, Permoserstraße 15, 04318, Leipzig, Germany. E-mail: sergio.gomez-olarte@ufz.de

Supporting Text

Supporting text 1. The LiNA study (**L**ifestyle and Environmental Factors and their **i**nfluence on the **N**ewborn **A**llergy Risk) is a prospective birth cohort conducted by the Helmholtz Centre of Environmental Research - UFZ in cooperation with clinical partners. It aims to investigate the impact of environmental factors on the development of children's immune system and the onset of allergic diseases, obesity, and behavioral disorders. A total of 629 mother-child pairs were recruited from March 2006 until December 2008 in Leipzig, Germany. Children and mothers were followed up annually until the age of 14 years by standardized questionnaires (ISSAC-based for health outcomes) and clinical appointments, during which blood, urine, and feces were collected. Thus far, environmental exposures, longitudinal follow-up of several immune parameters, endogenous metabolites, and gene expression analysis have been performed in children's blood and partially in maternal blood. Chemical exposure assessment during pregnancy has been conducted by targeted and non-targeted measurements in blood and urine samples.[1-3] Questionnaires were filled out by the mothers and contained information on several sociodemographic, lifestyle, psychological, and environmental factors as well as children's health outcomes. All participants gave written informed consent. The LiNA study was approved by the Institutional Review Board of the University of Leipzig (file reference No. 046-2006).

Supporting text 2. Leipzig is the most populous city in the German state of Saxony (formerly East Germany). The city had a population of 506,578 in 2006, when the LiNA study started, which has increased to 628,718 inhabitants as of 2024.[4] It is the eighth-largest city in Germany and is located in the Central German Metropolitan Region, about 150 km southwest of Berlin, the country's capital.

Supporting Tables

Table S1. Sociodemographic and lifestyle characteristics of LiNA cohort mother-child pairs with pesticide exposure assessment at baseline (pregnancy) and the 6-year follow-up.

Characteristic, n (%)	Pregnancy [a] n = 581	6-year follow-up [b] n = 387	*p*-value [c]
**Maternal age at delivery (years)**			0.649
< 25	61 (10%)	34 (8.8%)	
25-30	223 (38%)	146 (38%)	
30-35	197 (34%)	130 (34%)	
> 35	100 (17%)	77 (20%)	
**Smoking/ETS exposure during pregnancy**			0.074
No	470 (83%)	335 (87%)	
Yes	98 (17%)	50 (13%)	
Missing	13	2	
**Parental school education [d]**			0.239
Low	16 (2.8%)	5 (1.3%)	
Medium	128 (22%)	79 (20%)	
High	437 (75%)	303 (78%)	
**Breastfeeding (1-6 months)**			0.263
No	121 (22%)	73 (19%)	
Yes	417 (78%)	303 (81%)	
Missing	43	11	
**Family history of atopy**			0.978
None	192 (33%)	127 (33%)	
One parent	274 (47%)	185 (48%)	
Both parents	115 (20%)	75 (19%)	
**Child sex**			0.537
Male	303 (52%)	194 (50%)	
Female	278 (48%)	193 (50%)	

[a] LiNA cohort mothers with measurements of pesticides and their metabolites in urine and questionnaire data, n = 581 out of 622.

[b] Mother-child pairs at year 6 with complete outcome data.

[c] The Chi-square or Fisher's exact test (n < 5).

[d] Parental education defined by schooling years: 9 or fewer years (low), 10 years (medium), and 12 or more years (high).

Table S2. Distribution of pesticide and metabolite peak intensities measured in urine samples of the LINA cohort mothers and paired with their 6-year-old children (n = 387).

Compound	DR	GM	Min	Percentile					Max
				5th	25th	50th	75th	95th	
**Parent pesticide**									
Metalaxyl	0.90	1270366	187610	402920	755529	1164006	2007717	5048888	12413750
Carbetamide	0.57	344496	110515	149474	227315	329036	484946	962120	2927008
Terbuthylazine	0.45	215611	100679	108648	139098	195271	280879	784875	1666377
Imidacloprid	0.20	288459	149600	155023	210011	264976	320968	827975	2643546
Flonicamid	0.17	1086172	69116	190997	532492	1182724	2420594	5182573	11434016
**Pesticide metabolite**									
Hydroxy-isoproturon	0.84	2047844	204714	322923	629194	1494965	5638538	29027773	141951008
Dihydroxy-pyrimethanil	0.72	565423	118901	202270	339479	486930	796518	2382533	14872204
Hydroxy-simazine	0.46	322421	90101	141837	215994	297794	418327	1100428	4460982
Hydroxy-propamocarb	0.38	3695460	245184	351372	1084626	3285529	8916329	76557394	161967056
Fluazifop-desbuthyl	0.19	1645847	231956	469280	899686	1352976	2408504	13507945	50272536
Hydroxy-metazachlor	0.18	392895	144754	173737	266947	372449	540294	1036031	3604622

Abbreviations. DR: detection rate. GM: geometric mean; Min: minimum value; Max: maximum value.

Table S3. Results of logistic regression models exploring the association between prenatal exposure levels (log_2-transformed) to pesticides and allergies in 6-year-old children (n = 387). Exposures with values BDL were completed through multiple imputation (n = 20) using a truncated log-normal function, and the estimates were pooled based on Rubin's rule.

**Compound (log_2 scaled)**	**Asthma**				**Wheezing**				**Eczema**			
	**Unadjusted**		**Adjusted [a]**		**Unadjusted**		**Adjusted [a]**		**Unadjusted**		**Adjusted [a]**	
	**OR (95% CI)**	***p*-value**	**OR (95% CI)**	***p*-value**	**OR (95% CI)**	***p*-value**	**OR (95% CI)**	***p*-value**	**OR (95% CI)**	***p*-value**	**OR (95% CI)**	***p*-value**
**Parent pesticide**												
Metalaxyl	0.85 (0.63 - 1.13)	0.260	0.87 (0.65 - 1.17)	0.361	1.02 (0.89 - 1.17)	0.754	1.04 (0.91 - 1.19)	0.595	0.88 (0.75 - 1.03)	0.109	0.89 (0.76 - 1.05)	0.165
Carbetamide	1.24 (0.89 - 1.74)	0.199	1.24 (0.88 - 1.75)	0.228	1.00 (0.86 - 1.16)	0.964	1.01 (0.86 - 1.17)	0.944	0.92 (0.77 - 1.10)	0.337	0.91 (0.76 - 1.10)	0.322
Terbuthylazine	0.99 (0.65 - 1.50)	0.947	0.95 (0.62 - 1.47)	0.824	1.15 (0.96 - 1.38)	0.122	1.14 (0.94 - 1.37)	0.173	0.85 (0.68 - 1.07)	0.170	0.84 (0.67 - 1.07)	0.157
Imidacloprid	1.07 (0.60 - 1.90)	0.823	1.09 (0.60 - 1.98)	0.777	0.95 (0.72 - 1.25)	0.719	0.95 (0.72 - 1.26)	0.746	0.79 (0.56 - 1.13)	0.199	0.81 (0.56 - 1.16)	0.242
Flonicamid	1.01 (0.81 - 1.27)	0.909	0.98 (0.78 - 1.25)	0.895	1.06 (0.96 - 1.18)	0.221	1.05 (0.95 - 1.17)	0.337	0.97 (0.86 - 1.11)	0.689	0.95 (0.84 - 1.08)	0.464
**Pesticide metabolite**												
Hydroxy-isoproturon	0.86 (0.71 - 1.04)	0.123	0.85 (0.70 - 1.04)	0.108	0.95 (0.88 - 1.03)	0.250	0.95 (0.88 - 1.03)	0.232	1.02 (0.92 - 1.12)	0.727	1.01 (0.92 - 1.12)	0.787
Dihydroxy-pyrimethanil	1.35 (1.03 - 1.77)	0.032	**1.35 (1.02 - 1.79)**	**0.034**	1.05 (0.93 - 1.19)	0.398	1.05 (0.93 - 1.19)	0.406	1.03 (0.89 - 1.18)	0.707	1.02 (0.89 - 1.18)	0.747
Hydroxy-simazine	0.96 (0.69 - 1.33)	0.800	0.96 (0.68 - 1.35)	0.798	1.05 (0.91 - 1.21)	0.487	1.05 (0.91 - 1.21)	0.495	0.94 (0.79 - 1.11)	0.467	0.94 (0.79 - 1.11)	0.460
Hydroxy-propamocarb	1.05 (0.91 - 1.22)	0.519	1.09 (0.93 - 1.27)	0.279	0.95 (0.88 - 1.02)	0.145	0.95 (0.88 - 1.02)	0.153	1.00 (0.92 - 1.09)	0.984	1.00 (0.92 - 1.09)	0.926
Fluazifop-desbuthyl	1.11 (0.87 - 1.42)	0.415	1.14 (0.86 - 1.51)	0.354	1.15 (1.01 - 1.30)	0.037	**1.14 (1.01 - 1.30)**	**0.041**	0.99 (0.86 - 1.15)	0.942	0.99 (0.85 - 1.15)	0.847
Hydroxy-metazachlor	1.08 (0.68 - 1.72)	0.731	1.10 (0.68 - 1.79)	0.685	1.05 (0.85 - 1.31)	0.631	1.07 (0.85 - 1.33)	0.576	0.93 (0.71 - 1.21)	0.571	0.94 (0.71 - 1.25)	0.675

[a] Model adjusted for smoking/ETS exposure during pregnancy, breastfeeding up to 6 months, parental atopy history, parental education level, and child sex.

The odds ratios (ORs) in bold font are statistically significant: $p < 0.05$.

Abbreviations. BDL: below the detection limit.

Table S4. Output of logistic regression models examining the relationship between prenatal exposure levels (log_2-transformed) to pesticides and allergies in 6-year-old children (n = 387). Compounds with values BDL were imputed with LOD/√2.

Compound (log_2 scaled)	Asthma				Wheezing				Eczema			
	Unadjusted		Adjusted [a]		Unadjusted		Adjusted [a]		Unadjusted		Adjusted [a]	
	OR (95% CI)	*p*-value	OR (95% CI)	*p*-value	OR (95% CI)	*p*-value	OR (95% CI)	*p*-value	OR (95% CI)	*p*-value	OR (95% CI)	*p*-value
**Parent pesticide**												
Metalaxyl	0.84 (0.63 - 1.14)	0.255	0.87 (0.65 - 1.18)	0.363	1.02 (0.89 - 1.17)	0.747	1.04 (0.91 - 1.19)	0.572	0.88 (0.75 - 1.03)	0.104	0.89 (0.76 - 1.05)	0.169
Carbetamide	1.25 (0.91 - 1.72)	0.164	1.24 (0.89 - 1.73)	0.199	1.00 (0.87 - 1.15)	0.968	1.00 (0.87 - 1.15)	0.967	0.92 (0.78 - 1.09)	0.336	0.92 (0.77 - 1.09)	0.330
Terbuthylazine	0.97 (0.56 - 1.51)	0.901	0.93 (0.54 - 1.47)	0.784	1.20 (0.97 - 1.47)	0.092	1.18 (0.95 - 1.46)	0.131	0.82 (0.62 - 1.06)	0.145	0.82 (0.61 - 1.06)	0.146
Imidacloprid	1.09 (0.49 - 1.90)	0.801	1.12 (0.50 - 2.02)	0.733	0.96 (0.70 - 1.30)	0.772	0.96 (0.70 - 1.32)	0.818	0.71 (0.43 - 1.07)	0.141	0.74 (0.45 - 1.11)	0.183
Flonicamid	1.01 (0.76 - 1.26)	0.916	0.98 (0.72 - 1.24)	0.872	1.08 (0.96 - 1.20)	0.187	1.06 (0.95 - 1.19)	0.310	0.98 (0.85 - 1.11)	0.730	0.95 (0.82 - 1.09)	0.498
**Pesticide metabolite**												
Hydroxy-isoproturon	0.83 (0.66 - 1.03)	0.103	0.82 (0.65 - 1.02)	0.091	0.96 (0.88 - 1.04)	0.303	0.95 (0.87 - 1.04)	0.276	1.02 (0.92 - 1.12)	0.759	1.01 (0.91 - 1.12)	0.806
Dihydroxy-pyrimethanil	1.35 (1.03 - 1.79)	0.032	**1.36 (1.03 - 1.82)**	**0.032**	1.06 (0.93 - 1.20)	0.397	1.06 (0.93 - 1.20)	0.402	1.03 (0.88 - 1.19)	0.738	1.02 (0.88 - 1.18)	0.805
Hydroxy-simazine	0.95 (0.64 - 1.34)	0.786	0.95 (0.63 - 1.37)	0.788	1.05 (0.90 - 1.23)	0.509	1.05 (0.90 - 1.23)	0.519	0.93 (0.77 - 1.12)	0.471	0.93 (0.77 - 1.12)	0.460
Hydroxy-propamocarb	1.05 (0.88 - 1.23)	0.545	1.10 (0.91 - 1.30)	0.281	0.94 (0.87 - 1.02)	0.125	0.94 (0.86 - 1.02)	0.132	1.01 (0.91 - 1.10)	0.912	1.01 (0.91 - 1.10)	0.876
Fluazifop-desbuthyl	1.13 (0.83 - 1.43)	0.388	1.17 (0.84 - 1.56)	0.304	1.16 (1.01 - 1.35)	0.037	**1.16 (1.01 - 1.34)**	**0.043**	1.00 (0.84 - 1.17)	0.974	0.99 (0.82 - 1.16)	0.868
Hydroxy-metazachlor	1.08 (0.58 - 1.70)	0.759	1.11 (0.58 - 1.81)	0.708	1.06 (0.83 - 1.35)	0.631	1.08 (0.84 - 1.38)	0.568	0.89 (0.63 - 1.19)	0.462	0.90 (0.64 - 1.22)	0.538

[a] Model adjusted for smoking/ETS exposure during pregnancy, breastfeeding up to 6 months, parental atopy history, parental education level, and child sex.

The odds ratios (ORs) in bold font are statistically significant: $p < 0.05$.

Abbreviations. BDL: below the detection limit. LOD: limit of detection.

Supporting Figures

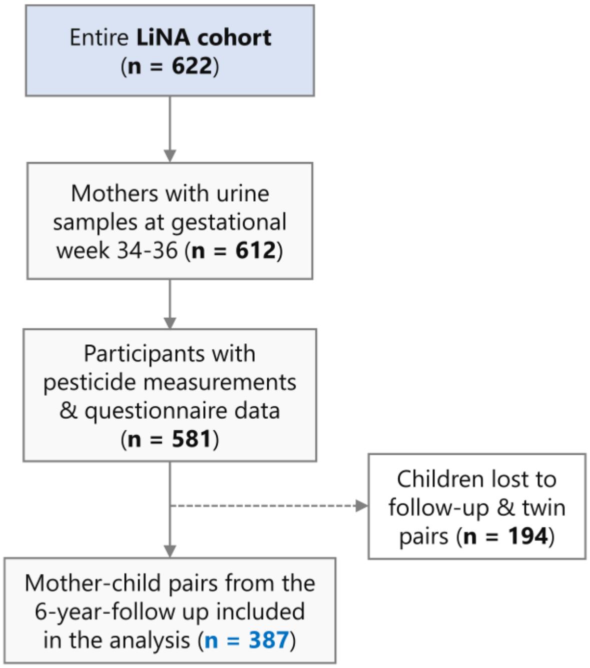

Figure S1. Flow chart of LiNA mothers (2006-2008) and paired 6-year-old children included in the study (n = 387).

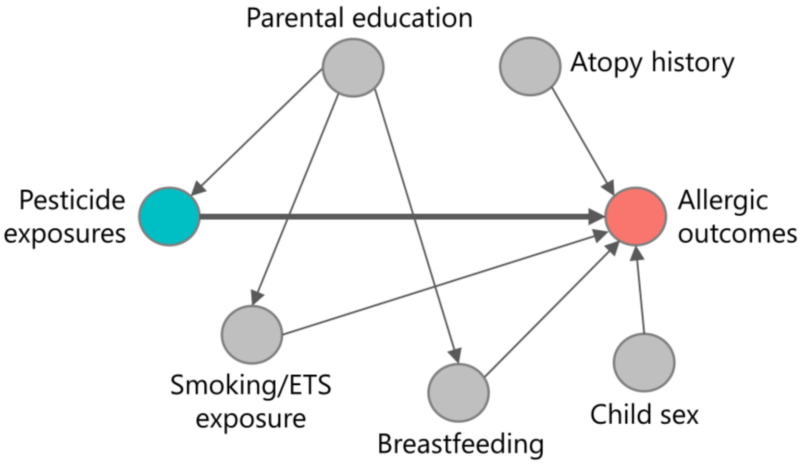

Figure S2. Directed acyclic graph (DAG) created to explore the causal pathway linking prenatal exposure to pesticides (light blue node) to allergic outcomes (red node), considering the effect of potential covariates (grey nodes). Regression models were adjusted for the covariates to estimate the total effect of the co-exposures on asthma, wheezing, and eczema.

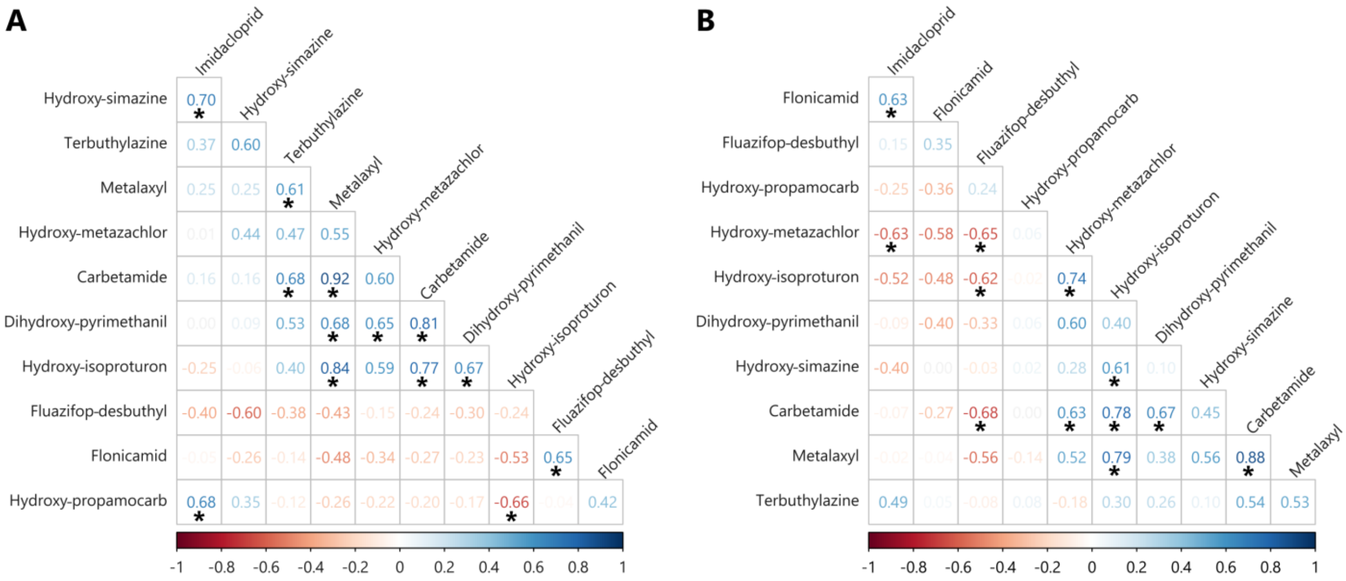

Figure S3. Pairwise Spearman's rank correlation matrices of chemical exposure levels in (**A**) the original dataset (LC-HRMS measurements) and (**B**) the LOD/√2-imputed dataset, where values BDL were imputed with the compound-specific minimum detection value divided by √2. In the color spectrum, blue and red shades show positive and negative correlations between the chemicals. The asterisk (*) denotes statistically significant correlations ($p < 0.05$).

References

(1) Herberth, G.; Pierzchalski, A.; Feltens, R.; Bauer, M.; Roder, S.; Olek, S.; Hinz, D.; Borte, M.; von Bergen, M.; Lehmann, I.; et al. Prenatal phthalate exposure associates with low regulatory T-cell numbers and atopic dermatitis in early childhood: Results from the LINA mother-child study. *J Allergy Clin Immunol* **2017**, *139* (4), 1376-1379 e1378. DOI: 10.1016/j.jaci.2016.09.034

(2) Huber, C.; Brack, W.; Roder, S.; von Bergen, M.; Rolle-Kampczyk, U.; Zenclussen, A. C.; Krauss, M.; Herberth, G. Pesticide residues and polyphenols in urine - A combined LC-HRMS screening to reveal intake patterns. *Environ Int* **2024**, *191*, 108981. DOI: 10.1016/j.envint.2024.108981

(3) Braun, G.; Herberth, G.; Krauss, M.; Konig, M.; Wojtysiak, N.; Zenclussen, A. C.; Escher, B. I. Neurotoxic mixture effects of chemicals extracted from blood of pregnant women. *Science* **2024**, *386* (6719), 301-309. DOI: 10.1126/science.adq0336

(4) Stadt Leipzig. *Einwohner und Bevölkerungsentwicklung* [Population and demographic development in Leipzig]. https://www.leipzig.de/buergerservice-und-verwaltung/unsere-stadt/statistik-und-zahlen/einwohner-und-bevoelkerungsentwicklung (accessed 2025-09-25).
